# Effects of New Btk-Based Formulations BLB1 and Lip on Aquatic Non-Target Organisms

**DOI:** 10.3390/biology13100824

**Published:** 2024-10-14

**Authors:** Sayda Dhaouadi, Rim El Jeni, Hazar Kraiem, Gul Ayyildiz, Cansu Filik-Iscen, Zeynep Yurtkuran-Ceterez, Balkiss Bouhaouala-Zahar

**Affiliations:** 1 Laboratoire des Biomolécules, Venins et Applications Théranostiques, Equipe NanoBioMedika, Institut Pasteur de Tunis, Université Tunis-El Manar, 13 Place Pasteur, BP74, Belvédère, Tunis 1002, Tunisia; sayda.dhaouadi@pasteur.utm.tn (S.D.); eljenirime@gmail.com (R.E.J.);; 2 Biyans Biyolojik ÜRÜNLER AR-GE DAN. SAN. TİC.LTD.ŞTİ., Mustafa Kemal Mah. Dumlupinar BLV.NO: 280: G İÇ KAPI NO: 1260, Çankaya 06530, Turkey; ayyildiz.gul@gmail.com (G.A.); zeynep.yurtkuran@biyans.com (Z.Y.-C.); 3 Department of Mathematics and Science Education, Faculty of Education, Eskisehir Osmangazi University, Eskisehir 26040, Turkey; cfilik@gmail.com; 4 Faculté de Médecine de Tunis, Université Tunis-El Manar, 13 Place Pasteur, BP74, Belvédère, Tunis 1002, Tunisia

**Keywords:** *Bacillus thuringiensis*, BLB1, Lip, Delfin^®^, ecotoxicological risk assessment, *Daphnia magna*, *Aliivibrio fischeri*

## Abstract

**Simple Summary:**

To mitigate the environmental-related harms caused by the continuous application of current pesticides, recent public health research is prioritizing the identification of new less harmful alternatives focusing on natural products such as biopesticides. The biosafety of two new *Bacillus thuringiensis subsp. Kurstaki*-formulated-based biopesticides BLB1 and Lip on the aquatic environment have been assessed. Their ecotoxicological effects were evaluated on two aquatic non-target organisms, *Daphnia magna* and *Aliivibrio fischeri*, in a comparative manner to Btk-Delfin^®^ industrial commercial product. Results pointed to the absence of significant acute effects on the motility/viability of *D. magna*. The survival/motility rate at 48 h of daphnids treated with BLB1 and Lip at 100 µg/ mL of δ-endotoxins is 60% and 80%, respectively. In contrast, the survival/motility rate of daphnids exposed to Delfin is only 15%. The bioluminescence of *A. fischeri*, exposed to BLB1 and Lip, in short-term tests, was not affected. In contrast, a hormetic effect was stimulated by BLB1 and Lip, clearly highlighting their potential use as new safe biological alternatives for plant pest protection. Further investigations are underway to characterize the environmental risk on terrestrial non-target organisms and lab animals, toward the full prediction of any BLB1- and Lip-associated exposure risks.

**Abstract:**

Integrated pest management based on the use of biopesticides is largely applied. Experimental bioassays are critical to assess biopesticide biosafety at the ecotoxicological level. In this study, we investigated the effects of the new *Bacillus thuringiensis subsp. kurstaki* (*Btk*)-formulated-based biopesticides BLB1 and Lip, efficiently tested in field assays (IPM-4-CITRUS EC project no. 734921) on two aquatic non-target organisms, precisely the water flea *Daphnia magna* and the bioluminescent bacteria *Aliivibrio fischeri*. Acute toxicity studies, carried out in a comparative manner with Delfin^®^ as the reference bioproduct and the lactose-based Blank formulation, show that no significant toxicity was observed up to 1 g/L. Our results indicated that BLB1- and Lip-formulated new bioproducts are far less toxic than the Delfin^®^ reference bioproduct.

## 1. Introduction

Chemical pesticides used for improving agricultural productivity have created numerous environmental issues, including the destruction of natural ecosystems, pesticide-tolerant diseases, and the genetic resistance of pest species (i.e., *Prays citri* and *Phyllocnistis citrella*). Among other things, product residues and toxicity to non-target organisms highly affect the biodiversity and organism life cycles [1]. Hence, it is noticeable that insect pest control could no longer be considered safe, dependent upon the utilization of chemicals.

Consequently, to circumvent the environmental-related harms caused by the continuous application of current insecticides [2], a recent public health concern research priority is to focus on the identification of new less harmful alternatives based mainly on natural products. Hence, growing attention has been directed toward exploring new non-toxic eco-friendly substances with pesticidal activity that would both act against particular ravager pests and prevent undesired health and environmental impacts (reviewed in [3,4]).

Based on their origin, biopesticides are classified into three major groups: (1) microbial, (2) biochemical, and (3) plant-incorporated protectants [5,6]. In particular, microbial biopesticides produced by soil microorganisms as active ingredients are the largest group of highly pest-specific broad-spectrum bioproducts. Owing to their origin, natural microbial biopesticides are safer and less harmful to non-target organisms (NTOs) than insecticides already on the market. The main advantages of such biological pest control active ingredients are that they are highly host-specific, have a long shelf life *in vivo*, are able to be mass-produced, and are easily transferable on an industrial scale [4,6].

*Bacillus thuringiensis* (Bt), which is an anaerobic gram-positive, crystalliferous, sporulating bacteria, is among the most used bacterial-based biopesticides for plant pest protection (PPP), usually inhabiting different environments such as soil, settled dust, and/or water. Bt strains have been shown to be toxic to specific phytopathogenic insects including lepidopterans as the main Bt target, coleopterans, dipterans, or nematodes, without any apparent toxicity to mammals. Bt larvicidal action is attributed to crystalline inclusion (or toxic protein crystal) produced during sporulation. Its life cycle comprises two main phases: (i) the exponential phase where the bacteria multiply and produce biomass (vegetative cells) and (ii) the sporulation phase where Bt strains begin to sporulate and synthesize protein crystals. Bt strains produce two types of toxins, Cry and Cyt proteins, which are also known as δ-endotoxins [7,8]. These endotoxins are produced as parasporal crystals, solubilized in the insect midgut, after activation through a well-known proteolysis process. Once activated, the endotoxins interact with gut receptors, creating pores in the intestinal cell membranes and leading to the death of the insect larvae [9].

Most of the commercial biopesticides distributed worldwide are *Bacillus thuringiensis* subsp. *kurstaki*-HD strain (Btk) based-formulations [10,11,12]. Recently, two new endemic strains, Btk-based Lip and BLB1, were isolated from Lebanese and Tunisian soils, respectively, exhibiting a higher efficiency than the HD-1 reference strain against the lepidopteran larvae *Ephestia kuehniella* [13,14]. Despite the Btk efficiency, experimental conditions using high doses of Btk were shown to affect some NTOs [15]. Therefore, predicting the environmental hazards of future end-use bioproducts must be routinely mandatory.

In this concern, this study aims first to assess the environmental risk related to BLB1- and Lip-based new formulations on aquatic organisms. For this purpose, the ecotoxicological effects of BLB1 and Lip were evaluated on two aquatic NTOs, explicitly an aquatic invertebrate (*Daphnia magna*) and a marine bacterium (*Aliivibrio fischeri*), in a comparative manner to a Blank formulation (devoid of Btk-endotoxins and spores) as well as Btk-Delfin^®^ industrial commercial product, used as “a gold standard” as it is one of the most commonly used commercial bioproducts of Btk. Delfin^®^ is a biological insecticide based on a selected strain of Btk, known as SA-11.

Targeting both *Daphnia magna* (*D. magna*) and *Aliivibrio fischeri* (*A. fischeri*), as two aquatic invertebrates, was essential as a first evaluation of BLB1 and Lip risks to the environment.

*D. magna* is widely used as a standard test species in environmental toxicology due to its short life cycle, fast reproduction, and high sensitivity to external contaminants (i.e., chemical pesticides, heavy metals, nanoparticles, and other manmade toxins) [16,17,18].

On the other hand, the marine bacteria *Aliivibrio fischeri (formerly Vibrio fischeri)* is one of the most common bio-indicators used for risk assessment in aquatic environments, based on the inhibition of luminescence produced by the bacteria in the presence of toxic substances [19].

In the present study, we assessed the acute toxicities of BLB1- and Lip-based newly formulated bioproducts on *D. magna* motility/viability and *A. fischeri* bioluminescence. This study allowed the investigation of the new bioproduct impacts on the behavioral changes and the bioluminescence at different concentrations and exposure times. Data showed, in both cases, insignificant attributed risks, clearly highlighting their potential use as new safe biological alternatives for PPP. Further investigations are underway to characterize the environmental risk on terrestrial NTO and lab animals, toward full prediction of any BLB1- and Lip-associated exposure risks.

## 2. Materials and Methods

### 2.1. Btk-Based Biopesticide Formulations

The biopesticides used in this study consisted of *Btk*-based formulations: Wettable new powder formulations of BLB1 and Lip-Btk isolates [13,14]. All batches freshly prepared and provided by Dr. Dietrich Stephan (Julius Kühn Institute, Darmstadt, Germany) were tested. The experiments were carried out in a comparative manner against *D. magna* and *A. fischeri*. Commercial Delfin^®^ WG 1 g/L of water with a potency of 32,000 IU/mL (Certis, Colombia, MD, USA) was used as a “gold standard” reference product and corresponded to *Btk* (6 × 10^7^ spores/mg). A Blank-formulated product containing all the additives except for the BLB1 or Lip endotoxins/spores’ active ingredients was also used as the control.

### 2.2. Acute Toxicity Assay on Daphnia Magna

To assess the aquatic environmental biosafety of Btk-based BLB1 and Lip new biopesticides, static acute toxicity tests lasting 48 h were conducted with *D. magna,* according to the established ISO and OECD guidelines (ISO 6341:2012 and OECD 202:2004) [20,21]. Briefly, *D. magna* (water flea) assays were performed according to the Standard Operational Procedures. The Daphtoxkit kit, which included *D. magna* ephippia, synthetic freshwater, and food (spirulina microalgae), was purchased from MicroBioTests (ref. TK33, Ghent, Belgium) and stored at 4 °C until use. First, reconstituted natural freshwater was prepared as recommended for the acute toxicity test with *D. magna*. Then, the standard freshwater was aerated for 15 min before being used for hatching the dormant ephippia. The contents of one ephippia vial were poured into a microsieve, rinsed with tap water to eliminate all traces of the storage medium, and then transferred into the hatching petri dish containing 15 mL pre-aerated standard freshwater before 72 h of incubation at 20–22 °C under continuous illumination of 6000 lux. The first set of experiments was performed with BLB1 and Lip bioproducts (Table S1) as well as the Delfin^®^ reference bioproduct, taking into consideration the δ-endotoxins concentrations (100, 50, 25, 12.5, and 6.25 µg/mL of δ-endotoxins), based on total protein concentrations. The δ-endotoxins concentration of each tested bioproduct was determined after alkaline protein solubilization and using Bradford assay, as previously described by Saadaoui et al. and Zouari et al. [13,22]. Untreated *D. magna* were considered as negative controls. The second set of experiments was carried out based only on weighting each formulated powder, without taking into consideration the δ-endotoxin protein proportions (1, 0.5, 0.25, 0.125, and 0.06 in g/L of each weighed powder).

Subsequently, a pre-feeding step, consisting of feeding neonates with spirulina algal suspension, took place over the span of 2 h. The test plates were prepared by filling 10 mL of the Btk-based biopesticide prepared solutions, corresponding to C_1_ to C_5_ concentrations (100, 50, 25, 12.5, and 6.25 µg/mL of δ-endotoxins or 1, 0.5, 0.25, 0.125, and 0.06 g/L of each weighed powder, respectively), into each testing well and 10 mL standard freshwater into each control well.

The *D. magna* used in the test were healthy individuals aged less than 24 h. Twenty daphnids (<6 h age <24 h) per concentration (5 daphnids per well, four replicates for each tested concentration) were exposed to different concentrations of lyophilized Delfin^®^, BLB1, Lip, and Blank in the assay medium. After that, the multiwell plates were incubated in total darkness at 20 ± 1 °C with a pH set to 7.5 ± 0.2. After 24 and 48 h, the total number of dead and immobile neonates was determined for each concentration, and the mean percent effect was calculated based on the following formula:

Percent Effect = (Observed Effect (number of immobile/dead Daphnia) × 100%)/20 (total number of Daphnia tested for each concentration).

The validity of the tests was confirmed when the number of dead and immobile organisms did not exceed 10% of controls. The effect criteria were immobility and mortality. The number of affected (immobilized and dead) organisms in each well was determined in a short-term exposure and static system at 24 and 48 h, and EC_50_ values were calculated (Table 1). Individuals who died or showed morphological abnormalities were observed under a light microscope.

The data expressed as EC_50_ were transformed into Toxic Units (TUs) to reveal the direct relationship between toxic effects and the test system used. TUs were calculated according to Equation (1).

TU = [1/EC_50_] × 100 (1)

### 2.3. Acute Toxicity Assay on Aliivibrio Fischeri

Bioluminescence inhibition in the marine bacterium *A. fischeri* was evaluated using freeze-dried bacteria from the Microtox test (Microbics Corp., Carlsbad, CA, USA). The freeze-dried photobacteria *A. fischeri* (1243-157) were reconstituted by pouring one vial of cooled (4 °C) reagent diluent (1243-110). Subsequently, the rehydrated *A. fischeri* bacteria were equilibrated at 4 °C for at least 30 min and then stabilized at 15 °C (as specified in ISO 11348 [23]) for another 30 min. The Btk-based BLB1, Lip, and Delfin^®^ as well as the Blank formulations were prepared by adding 2.0 g of wettable powder into 8.0 mL sample diluent (ref. 1243-125). Samples were homogenized; pH was adjusted to 7.0 ± 0.2 and diluted (from 1/2 to 1/32 serial dilutions). The assay was performed in cuvettes, 200 µL of each sample was transferred into each cuvette and, inside the luminometer, 200 µL of bacterial suspension was automatically dispensed onto the sample. Measurements were performed using a Microtox luminometer (Model 500 Analyzer, Model 500 Microtox^®^, Strategic Diagnostics, Newark, DE, USA). The luminometer allowed for the dispensing of bacterial reagent, continuous mixing of the sample, and simultaneous measuring of the luminescence. The bioluminescence was measured after 5 min during 15 s. After 15 min of incubation, the bioluminescence was measured again for 15 s.

### 2.4. Statistical Analysis

All experiments were performed at least twice, with four replicates for each condition. The EC_50_ values were calculated using the REGTOX: macro-excel^TM^ version for all assays. Viability (mean number of alive daphnids) was assessed between groups with an unpaired student’s *t*-test or one-way ANOVA with Tukey HDS or Dunett tests using GraphPad Version 5.1, with *p* < 0.05 being considered a significant difference.

## 3. Results

### 3.1. Sensitivity Response of D. magna toward Various Concentrations of BLB1, Lip, and Delfin^®^

The first experiments were carried out with a particular focus on Btk-δ-endotoxins concentrations (100, 50, 25, 12.5, and 6.25 µg/mL serial dilutions based on total protein concentrations) within the new Btk biopesticide developed formulations versus the Delfin reference product. The δ-endotoxins concentration of each tested bioproduct was determined after alkaline protein solubilization followed by the Bradford assay (Table S1).

**Figure 1:**
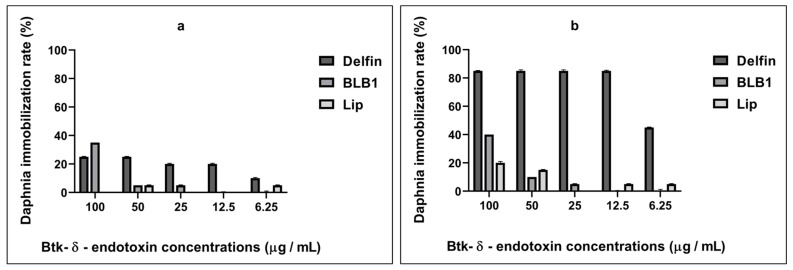
Dose–response relationships between Btk-δ-endotoxin concentrations (100 µg/mL to 6.25 µg/mL) and the immobilization rate of *D. magna* after 24 h (**a**) and 48 h (**b**) of exposure. Data expressed relative to mean values with respect to unexposed controls.

**Figure 2:**
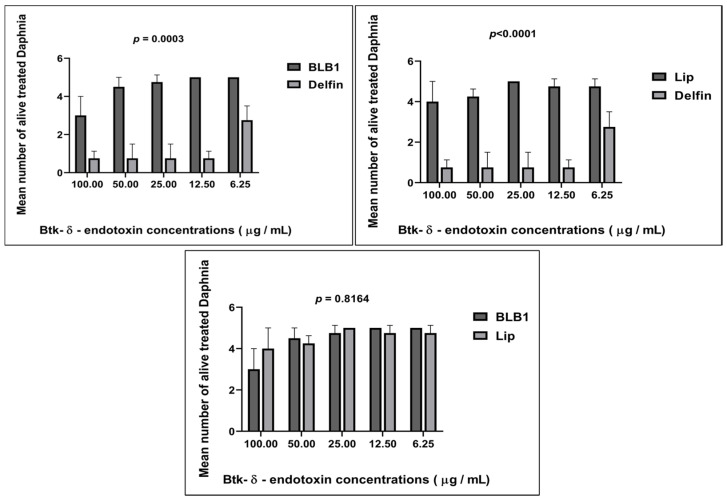
Viability of the treated *D. magna* after exposure to the Btk biopesticides, tested at different δ-endotoxin concentrations (endpoint assessment at 48 h of alive treated daphnids). Data expressed relative to mean value (the mean number of alive treated *D. magna*) in a dual comparative manner. Bars represent the mean ± SE. Unpaired student’s *t*-test was performed.

Interestingly, a dose–response relationship between Btk-δ-endotoxin concentrations and *D. magna* motility was noticeable. In the presence of Btk-BLB1 product, there was no immobilization nor death recorded at 6.25 µg/mL and 12.5 µg/mL product concentrations (C_5_ and C_4_ concentrations, respectively) following 48 h of exposure (Figure 1 and Figure 2). The daphnids’ immobilization was detected starting from exposure to 25 µg/mL of the Btk-δ-endotoxins (C_3_). At the highest C_1_ concentration of 100 µg/mL, the survival/motility rate at 48 h was 60% (Figure 1). In the presence of Btk-Lip product, an immobilization rate of 5% was observed at 6.25 µg/mL and 12.5 µg/mL bioproduct concentrations (C_5_ and C_4_, respectively). At the highest concentration (C_1_) of 100 µg/mL, the survival/motility rate at 48 h was 80% (Figure 1 and Figure 2). In contrast, the immobilization rate of daphnids treated with the Delfin^®^ reference bioproduct was 45% at the lowest tested concentration (C_5_) of 6.25 µg/mL. At the highest concentration (C_1_) of 100 µg/mL, the survival/motility rate at 48 h was only 15% (Figure 1). This assay clearly demonstrated that the crustacean *D. magna* is sensitive to Delfin^®^, showing a dose-dependent curve of immobilization with an EC_50_ of 50.662 µg/mL after 48 h of exposure (Table 2). In contrast, *D. magna* sensitivity to BLB1 and Lip δ-endotoxins is only significant at the highest tested concentration (C_1_, 100 µg/mL) (Figures S1 and S2), compared to data recorded with the Delfin^®^ reference bioproduct (Figure 1 and Figure 2).

The 48 h EC_50_ corresponds to 96. 86 mg/L and 91.25 for Lip and BLB1 newly formulated bioproducts, respectively. Notably, the Delfin^®^ bioproduct is about two-fold more toxic (50.662 mg/L) than Lip and BLB1 (Table 2).

**Figure 3:**
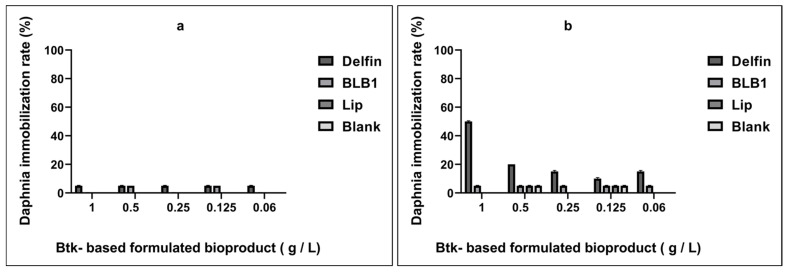
Dose–response relationships between Btk biopesticide-formulated bioproducts (1 g/L to 0.0625 g/L) and the immobilization rate of *D. magna* after 24 h (**a**) and 48 h (**b**) of exposure. Data expressed relative to mean values in respective unexposed controls.

**Figure 4:**
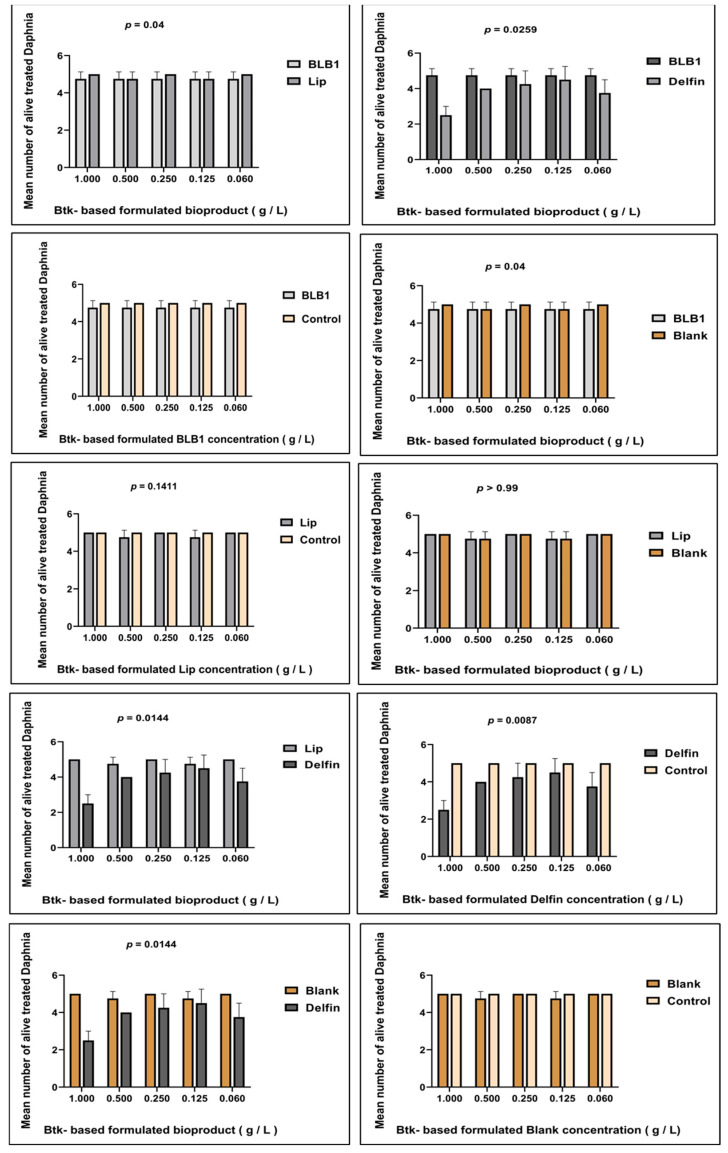
Viability of *D. magna* after exposure to various Btk biopesticides tested at different concentrations (endpoint assessment = 48 h). Data expressed relative to mean values (the mean number of alive treated *D. magna*) in a dual comparative manner. Bars represent the mean ± SE. Unpaired student’s *t*-test was performed.

In order to assess the toxicity of the formulated bioproducts, as part of the Quality-Control (QC) process, the set of experiments was reiterated in a blind comparative manner, taking into consideration only the weighted powders without considering the comprised Btk-δ-endotoxin yields and spores. The bioproduct powders of BLB1, Lip, and Delfin were weighted equally (C_1_ concentration). Following 24 and 48 h of exposure, the effects on daphnids were assessed by monitoring immobilization/mortality numbers comparatively to the controls (Figure 3 and Figure 4). No significant change in the daphnids’ motility was observed in the control group as well as in the tested BLB1 and Lip bioproducts, at the different concentrations. In Figure 3, the percentages of immobilized *D. magna* for each tested Btk biopesticide concentration are reported. Interestingly, the highest immobilization rate (50%) was reached only with the Delfin^®^ bioproduct at 1 g/L. The immobilization rate increased tenfold over time, from 24 to 48 h of exposure.

Conversely, after 48 h of exposure, the immobilization/mortality effects did not exceed 5% for Daphnia neonates treated with Btk-based formulated BLB1 and Lip bioproducts. Altogether, the results are similar to the Blank formulation at different concentrations. The results indicated that 100% of daphnids in the control group did not exhibit abnormal swimming behavior or mortality. Moreover, dose–response effects were only recorded when the reference Btk-Delfin^®^ whole product was tested. However, no dose–response results were recorded with the new Btk-based formulated BLB1 and Lip bioproducts. The mean of alive daphnids treated with BLB1 was significantly different from that of daphnids exposed to Lip (*p* = 0.04) (Figure 4). Increasing concentrations of BLB1 and Lip did not significantly lead to toxic effects on *D. magna* (Figure 3). The results indicate that BLB1 and Lip do not cause acute toxicity for the *D. magna* NTO at concentrations up to 1 g/L (Figure 4 and Figure S3).

Based on the established REGTOX macro-excel^TM^ analysis software, after 48 h of exposure, the EC_50_ corresponds to 1263.884 mg/L and 1256.741 mg/L for BLB1 and Lip, respectively (Table 3). The lowest EC_50_ was determined for the Delfin^®^ (1013.39 mg/L). Effective concentrations resulting in a lethality rate of EC 5%, 10%, 15%, 20%, and 50% are detailed in Table 3.

**Figure 5:**
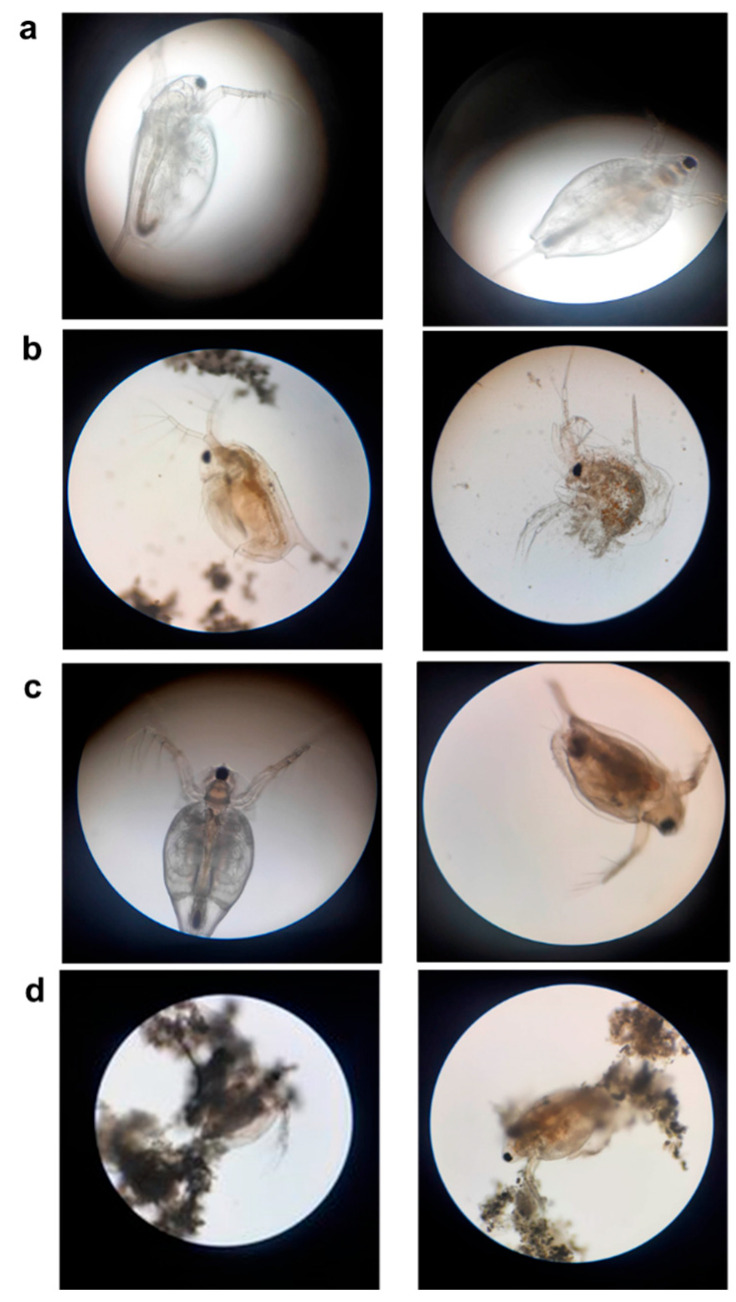
Appearance of *D. magna* treated with three different Btk-based formulated biopesticides. (**a**) Non-treated *D. magna.* (**b**) Dead *D. magna* treated and exposed to Delfin^®^-WG. (**c**) Alive (**left**) and dead (**right**) daphnids exposed to BLB1. (**d**) Daphnids stuck to the insoluble Lip, causing their immobility.

Figure 5b presents dead daphnids exposed to Delfin. One of them (right) has lost its carapace, which is an indicator of severe toxicity. Figure 5c presents daphnids exposed to BLB1. The right figure shows a dead daphnid with a dark gut. The dark coloration of the *Daphnia* gut is indicative of the accumulation of the BLB1 substance or its metabolites in the digestive tract or hemolysis due to physiological stress.

### 3.2. Sensitivity of A. fischeri to New BLB1, Lip, and Delfin^®^

**Figure 6:**
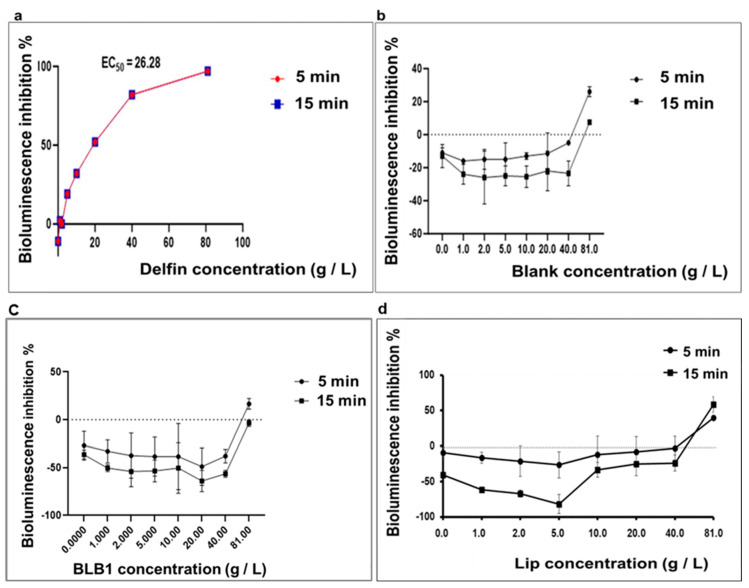
Concentration–response curves of the tested Btk-based biopesticides at 5 min and 15 min in the acute *A. fischeri* assay. The influence of exposure to Delfin (**a**), Blank control (**b**), BLB1 (**c**) and Lip (**d**) on the bioluminescence of *A. fischeri* was assessed.

The effects of BLB1- and Lip-formulated bioproducts on the bioluminescence of *A. fischeri* bacteria were studied after 5- and 15-min exposures at varying concentrations to establish dose–response curves (Figure 6).

After 15 min of *A. fischeri* exposure, no inhibition of bioluminescence was observed. The inhibition rate was expressed in negative values, revealing that the tested Btk samples (BLB1 and Lip) were less toxic than the control. These stimulatory responses at low doses are related to hormetic dose/concentration responses.

A hormetic dose–response relationship is characterized by the width of the stimulatory zone, the zero-equivalent point (ZEP), and the maximum stimulation effect (E_m_). The concentrations at the ZEP point (EC_0_) were approximately 80, 81, and 60 g/L for Blank, BLB1, and Lip, respectively (Figure 6b–d). Considering the recorded dose–response curves, the concentrations of the E_m_ (EC_m_) were approximately 40, 20, and 5 g/L for Blank, BLB1, and Lip (Figure 6b–d). Up to 40 g/L, the two tested Btk biopesticides (BLB1 and Lip), as well as the Blank formulation, could elicit *Aliivibrio* luminescence stimulation. However, *A. fischeri* exhibited very high sensitivity towards the Delfin reference bioproduct (Figure 6a). These results clearly indicated that *A. fischeri* presents tolerance towards Btk-tested biopesticides BLB1 and Lip compared to the Delfin^®^ reference bioproduct.

## 4. Discussion

Public health policy matters and environmental considerations dictate that proof of safety to NTOs should be documented before any pest control agent application in actual pest control programs. Microbial control agents are not exempt from this mandate. Therefore, extensive environmental and biological impact data must be necessarily provided before microbial agents (i.e., *Bt*) can be approved for public use.

In fact, previous studies showed that some NTOs are affected either by single or repeated *Bt* treatments. Saadaoui et al. (2009) determined that BLB1 crystal protein toxicity resulted in an LC_50_ of 70.32 ng of toxin per mg of flour against third instar *Ephestia kuehniella* with confidence limits of (31.6–109.04 ng) [13]. Furthermore, BLB1 bioactive components issued from the recovery ultrafiltration process presented a lethal LC_50_ of 194.00 µg/g of wheat semolina [24]. The Btk-Lip and HD-1 strains present an LC_50_ of 33.27 and 128.61 μg toxin/g semolina, respectively [14].

We tested Btk-based biopesticides on aquatic NTOs as a part of the QC process. It is a well-known fact that pesticides and their metabolites are transported from a targeted to a non-targeted area via adsorption, leaching, volatilization, or surface runoff [25,26].

In the environment, pesticides undergo transformations and can be moved between ecosystems in their initial form or as derived metabolites, which often exhibit higher toxicity than the initial compounds. Such forms of pesticides can penetrate soil, water, and air, as well as animal feed and food products, posing a direct threat to living organisms [27]. Biopesticides are not exempt from this metabolite transformation. In fact, it has already been demonstrated that *Bacillus* proteins can be dispersed in aquatic environments adjacent to crop fields through spraying *Bacillus* formulations [28,29]. The frequency of application and the dosages applied could play a role in the persistence of *Bacillus* crystals in the environment, which can affect NTOs as well as the food web because some predators that feed on these drifting insects could be also affected. As an example, Boisvert and Boisvert (2000) found that Bti crystals could be adsorbed rapidly onto vegetation and remain very toxic for 22 weeks [11].

The choice of *D. magna* and *A. fischeri* was based on their well-established roles as non-target aquatic ecotoxicity indicator organisms [11,29,30,31]. *D. magna* have been used extensively for the identification of pesticide effects on non-target aquatic invertebrates due to their high sensitivity.

Zooplankton play an important role in the aquatic food chain as the main consumer of bacteria, single-cell algae, and organic detritus and the main food source for higher trophic levels, including fish. Changes in their abundance, diversity, or distribution can impact an entire aquatic ecosystem [29]. Interestingly, zooplankton are highly sensitive to many contaminants and are thus used as a bioindicator to monitor changes in water quality.

This study provides, for the first time, an assessment of the risk from aquatic environmental exposure to the new Btk-based BLB1- and Lip-formulated biopesticides and shows that these new bioproducts might not cause acute toxicity toward the two main commonly affected species, hereby considered non-target aquatic organisms.

The experimental results pointed to the absence of acute effects on the motility/viability of *D. magna* and the bioluminescence of *A. fischeri* exposed to the two new Btk-isolated strains in short-term tests. To our knowledge, this ecotoxicological reached an endpoint of No-Observed-Adverse-Effect Level (NOAEL), attributed to both newly formulated BLB1- and Lip Btk-based biopesticides, is the first assessed study.

Given that the insecticidal property of *Bt* is attributed to δ-endotoxins, it was worth conducting an initial assessment strategy, considering the Btk-δ-endotoxins concentration. Results showed that Delfin^®^ is the most acutely toxic to *D. magna* with an EC_50_ value of 50.6628 mg/L. Commercial Delfin^®^ reference bioproduct displayed a clear dose-dependent effect on Daphnia motility/viability, whereas BLB1 and Lip present higher EC_50_ values under the same experimental conditions. At the highest tested concentration (C_1_ = 100 µg/mL), immobilization rates of 40 and 20% were determined when *D. magna* neonates were exposed to BLB1 and Lip, respectively. In a distinct study, Chen and co-authors demonstrated that the δ-endotoxin protein (Cry1C) tested at a concentration of 500 µg/L did not engender significant effects on the development, reproduction, and reproductive parameters of the *D. magna* after 21 days of exposure [29].

The second trial did not focus on the concentrations of δ-endotoxins per bioproduct, but instead used equal amounts of tested Btk biopesticides, to evaluate the synergetic activity combining δ-endotoxins and spores. The concentration range was chosen based on the reference bioproduct providers’ operational recommended dosages. Assorted growth inhibitory concentrations (EC) were determined (Table 3). In addition, we performed a comparative study with the Btk-standard reference formulation (Delfin^®^-WG, 32,000 IU) (Table 4).

Results showed that the immobilization effect did not exceed 5% for Daphnia neonates exposed to the different Btk-based formulated BLB1 and Lip as well as to the Blank formulation. However, the immobilization rate of daphnids exposed to the Delfin^®^ reference bioproduct reached 50%. This finding is consistent with an earlier study that showed that Bti-based bioproduct (i.e., *Bt* subspecies *israelensis*) tested at 5000 ppm (parts per million), (5 g/L) caused a *D. magna* mortality rate of 80% [11]. In a previous study, a Brazilian *Btk*-isolated strain was tested on the crustacean *Daphnia similis* in a ranging concentration up to 1.5 × 10^6^ CFU/mL. The results showed that no toxic effect was recorded [32].

Daphnid swimming behavior is a common biomarker widely used in the assessment of a bioproduct toxicity effect [33,34]. The results demonstrated that BLB1 and Lip did not cause abnormal swimming behavior (swimming speed and turning ability) in the 48-h short-term exposure, suggesting that the sensory and locomotory activities of daphnids were not affected. However, their potential risk of sublethal adverse effects, including growth, fecundity, and reproduction, must be assessed over prolonged exposure periods. Through a chronic toxicity test, a biochemical analysis might be conducted to assess the influence of BLB1 and Lip on the SOD, POD, and CAT enzyme activities of *D. magna.* These antioxidant enzymes are important to protect organisms against the peroxidation system and maintain the redox state of the cell. SOD is the first line of cell defense, as it neutralizes superoxide anions (O_2_^−^) to yield hydrogen peroxide and molecular oxygen [29,35].

Since *D. magna* is sensitive to disturbances that occur in aquatic environments, microscopic observations were conducted, showing that the whole carapace of the Daphnia was removed as a result of Delfin^®^ toxicity (Figure 5). However, only two daphnids treated with Blank or Lip were immobilized as they were stuck and adhered to the spheroid Blank/Lip solutions. We suggest that the immobilization was likely to be due to the plugging of the gills rather than the toxic effects caused by Lip and Blank. This observation leads us to suggest that the immobilization state is not related to Blank/Lip toxic effects but to their low aqueous solubility. Indeed, the hazardous potential of biopesticides is not limited to the toxic effects of bioactive compounds but is also likely based on their uptake and elimination kinetics and their bioavailability, dispersion, or accumulation in the environment [36]. Furthermore, biopesticide formulation issues, such as solubility, have been reported, which may influence the perceived toxicity and hazard potential of biopesticides [37].

Recently, nanobiopesticides were developed based on the embedding of the bioactive ingredients into a nanocarrier. This approach might resolve problems related to formulation such as low aqueous solubility, high volatilization rates, and susceptibility to oxidation [38]. By encapsulating the bioactive ingredients in nanoparticles, they can be dispersed more effectively in aqueous solutions, improving their solubility as well as their bio-availability.

Moreover, light microscopic images showed the accumulation of the BLB1 and Lip Btk bioactive ingredients in the daphnids’ gut after 48 h of exposure. Hence, BLB1 and Lip accumulation did not cause abnormal swimming behavior. However, BLB1 and Lip could be retained in the gut and produce a risk of chronic toxicity over a prolonged exposure period, which needs further investigation for better understanding.

To assess the potential impacts of Btk-based BLB1 and Lip on aquatic invertebrates, additional tests were performed with *A. fischeri*. We observed an interference, due to particle Btk bioactive-ingredient-related turbidity, with the *A. fischeri* luminescence signal. Interestingly, a clear hormesis phenomenon was observed, especially for Lip.

Hormesis is a dose–response relationship phenomenon characterized by low-dose stimulation and high-dose inhibition [39]. It is a criterion that is becoming a central concept in toxicology [40]. Hormesis was frequently observed in the toxicity tests on luminescent bacteria [41,42].

Low Delfin^®^ concentrations greatly decrease bioluminescence (Figure 6a). Conversely, low concentrations of Blank, BLB1, and Lip increase luminescence intensity compared to the control (Figure 6b–d). This phenomenon associated with hormesis has been already described in previous studies [43]. The molecular mechanism underlying hormesis has also been previously detailed [39,44]. The authors showed that antibiotics tested at low concentrations, acting as autoinducers, can activate the gene expression of luminescent proteins. However, as the doses increase, the progressive binding of antibiotics to dihydropteroate synthase, which inhibits the synthesis of folic acid, causes toxic effects [40]. Hence, in this study, evidence has been provided showing that low doses of the different Btk biopesticides (BLB1 and Lip) exerted a hormetic effect on quorum sensing, which is a regulatory mechanism of *A. fischeri*. These results reveal that *D. magna* is more sensitive than *A. fischeri* to BLB1 and Lip new bioproducts.

The investigation of Lip and BLB1 impacts on terrestrial NTOs is currently underway.

Although these studies show important information on BLB1 and Lip effects, they may not reflect actual environmental exposure that may occur over multiple generations. Conducting chronic toxicity tests that could be extended for two generations could yield additional insight into more ecologically relevant effects due to biopesticide exposure.

## 5. Conclusions

This study presents a novel assessment of the risk posed by aquatic environmental exposure to the new Btk-based BLB1- and Lip-formulated biopesticides.

Based on the available data, many results are difficult to compare due to the diversity of measurement units used in the different research studies. Nevertheless, we manage, based on these findings, to demonstrate that BLB1 and Lip new formulations presented low toxicity towards aquatic NTOs compared to the concentrations used to test other Bt subspecies. We demonstrate that the new Btk-based biopesticides BLB1 and Lip have an insignificant risk of toxicity to an aquatic environment. More studies are further required to fully understand their ecological impact, particularly on terrestrial NTOs and on potential chronic toxicity, in order to ensure safer incorporation of these biopesticides into agricultural practices.

## Figures and Tables

**Table 1:** Properties of the performed test protocols. (*): Daphtox and Microtox are trademarked brands.

Test	Trophic Level	Group of Organisms	Type of Test	Test Duration	Test Criterion	Test Principle
Microtox * (*Aliivibrio fischeri*)	Decomposer	Bacteria	acute	15 min	Bioluminescence inhibition	Measure of luminescence reduction with luminometer
Daphtox * (*Daphnia magna*)	Primary consumer	Crustaceans	acute	48 h	Immobility/Mortality	Counting of immobilized/dead and alive crustacean

**Table 2:** Effective concentration values (EC in mg/L) of the three tested bioproducts, based on the Btk-δ-endotoxins concentrations. The toxicity units (TUs) of the three Btk bioproducts were assessed against *D. magna*.

Bioproduct		EC_5_	EC_10_	EC_15_	EC_20_	EC_50_	TU
Delfin^®^	Optimal	14.855110814	20.280975727	24.592538668	28.43358065	50.66287994	1.97
	Average	14.855111017	20.280975848	24.592539295	28.43357995	50.6628789	
	Median	14.855111122	20.280975341	24.592538833	28.4335804	50.66287994	
BLB1	Optimal	53.936655407	61.635712778	66.943755933	71.2397125	91.25	1.095
	Average	53.936656564	61.635710299	66.943757236	71.2397173	91.25000238	
	Median	53.936655951	61.635711669	66.943756103	71.23971558	91.25	
Lip	Optimal	70.335694387	76.286681764	80.22085677	83.31619615	96.86585999	1.032
	Average	70.335693657	76.286680996	80.22085577	83.31619203	96.86586261	
	Median	70.335693359	76.286682128	80.22085571	83.31619263	96.86585999	

**Table 3:** Effective concentration values (mg/L) of the three Btk-based formulated bioproducts and the Blank formulation. Their toxicity units (TUs) were assessed against *D. magna*.

Bioproduct	EC_5_		EC_10_	EC_15_	EC_20_	EC_50_	TU
Delfin^®^	Optimal	932.3414516	952.2753551	964.8300967	974.3915958	1013.395661	0.09867
	Average	968.3056653	993.0435114	1008.840638	1020.989531	1071.710124	
	Median	914.4064941	937.2280884	954.8158569	967.4196167	1012.115662	
BLB1	Optimal	1216.949498	1228.692693	1236.019997	1241.565502	1263.884835	0.07912129
	Average	1360.461129	1373.301712	1381.325891	1387.405276	1411.930563	
	Median	1216.949341	1228.692505	1236.019775	1241.565308	1263.884644	
Lip	Optimal	1211. 38623	1222. 83924	1229.83385	1235.24384	1256.74177	0.07619
	Average	1211.4529137	1222.772556	1229.76717	1235.17715	1256.67509	
	Median	1211.5196	1222.77256	1229.70048	1235.11047	1256.60841	
Blank	Optimal	1249.645277	1265.305134	1275.098841	1282.522459	1312.5	0.07619
	Average	1249.645233	1265.305161	1275.09892	1282.52244	1312.5	
	Median	1249.645264	1265.305176	1275.098877	1282.522461	1312.5	

**Table 4:** Comparative sensitivity of *D. magna* and *A. fischeri* to BLB1, Lip, and Delfin^®^, ranking from the highest to the lowest toxicity. Analysis conducted using unpaired Student’s *t*-test. (*): significance is defined as *p* ≤ 0.05.

Species	Ranking	Significance (*)
*Daphnia magna*	Delfin^®^ > BLB1 > Lip	Delfin versus BLB1 (*p* = 0.0259) Delfin versus Lip (*p* < 0.0001)
*Aliivibrio fischeri*	Delfin^®^ > Lip > BLB1	Delfin versus BLB1 (*p* = 0.0002) Delfin versus Lip (*p* = 0.0029)

## Data Availability

Supporting research data can be shared through specific requests by email.

## Supplementary Materials

The following supporting information can be downloaded: https://www.mdpi.com/article/10.3390/biology13100824/s1, Figure S1: Viability of the treated *D. magna* after exposition to the various Btk biopesticides tested at different δ-endotoxins concentrations; Figure S2: immobilization rate of Daphnia magna exposed to the various Btk biopesticides tested at different δ-endotoxin concentration; Figure S3: viability of the treated *D. magna* after exposition to various Btk-biopesticide-formulated bioproducts, tested at different concentrations. Table S1: Endotoxin concentrations based on Bradford assay.

## Abbreviations

*Bt*, *Bacillus thuringiensis*; Btk, *Bacillus thuringiensis subsp. kurstaki*; CFU, colony-forming unit; EC, effective concentration; g, gram; h, hour; IU, international unit; ISO, the International Organization for Standardization; LC_50_, lethal concentration 50%; L, liter; µg, microgram; mg, milligram; min, minute; NTOs, non-target organisms; OECD, the Organization for Economic Co-operation and Development; ppm, parts per million; s, second; SE, standard error; TU, toxic unit; WG, Water-Dispersible Granules; ZEP, zero equivalent point.
