# Supplementary material for: Effects of New Btk-Based Formulations BLB1 and Lip on Aquatic Non-Target Organisms
Source: Biology (Basel). 2024 Oct 14;13(10):824. doi: 10.3390/biology13100824 (PMC11505242; doi:10.3390/biology13100824)
Supplement: Supplementary file 1 [file biology-13-00824-s001.zip › biology-3134317-supplementary.pdf]

Table S1. Endotoxin concentrations based on Bradford assay

Btk-biopesticide	Endotoxin concentrations
**BLB1**	1992,941 µg/100mg
**Lip**	1500,784 µg/100mg
**Delphin**	7045,882 µg/100mg

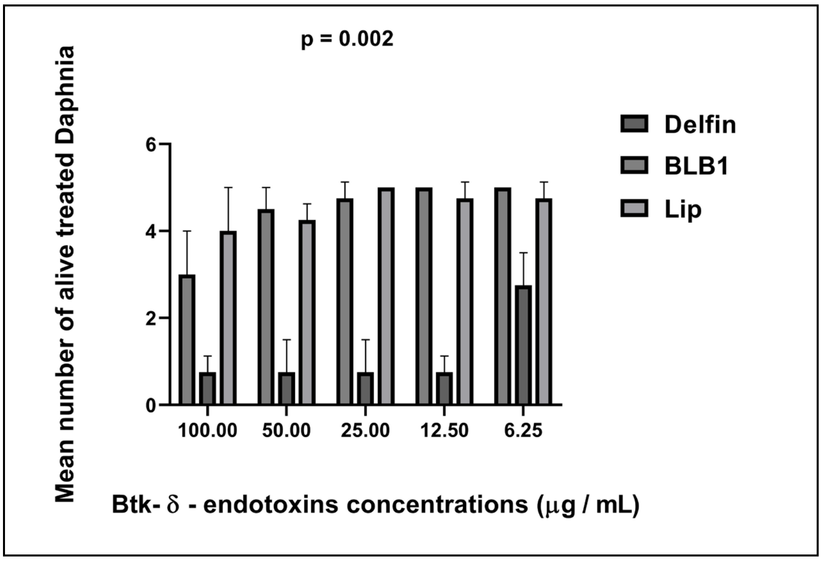

*

δ- endotoxins concentration (µg/mL)

C1	100
C2	50
C3	25
C4	12.5
C5	6.25

Figure S.1. Viability of the treated D. magna after exposition to the various Btk-biopesticides, tested at different δ - endotoxins concentrations (Endpoint assessment at 48 h of alive treated Daphnia). Data expressed relative to mean value. Bars represent the mean ± SE. The significance has been assessed using one-way ANOVA with Tukey test.

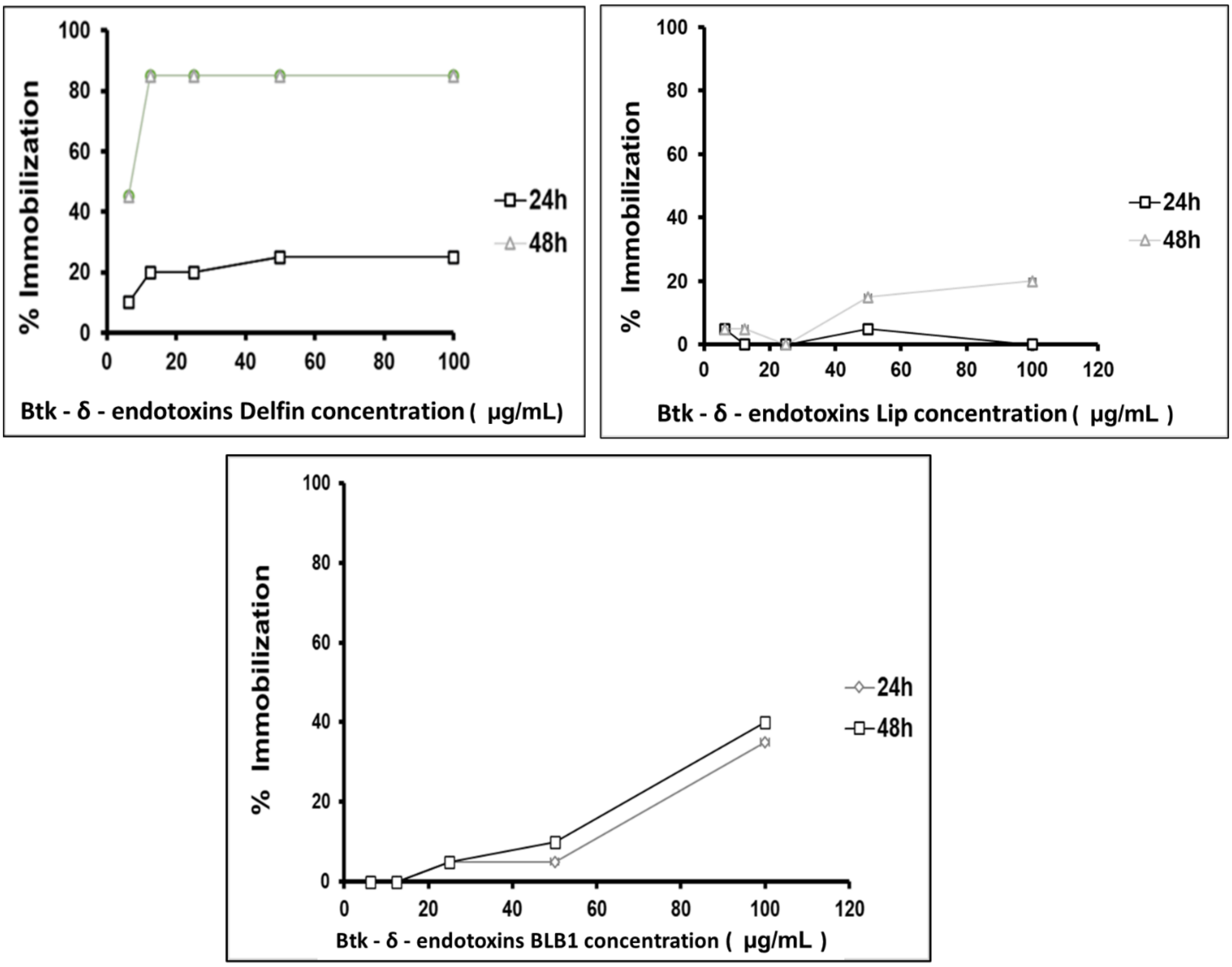

Figure.S2. Immobilization rate of D. magna exposed to the various Btk-biopesticides, tested at different δ - endotoxins concentration.

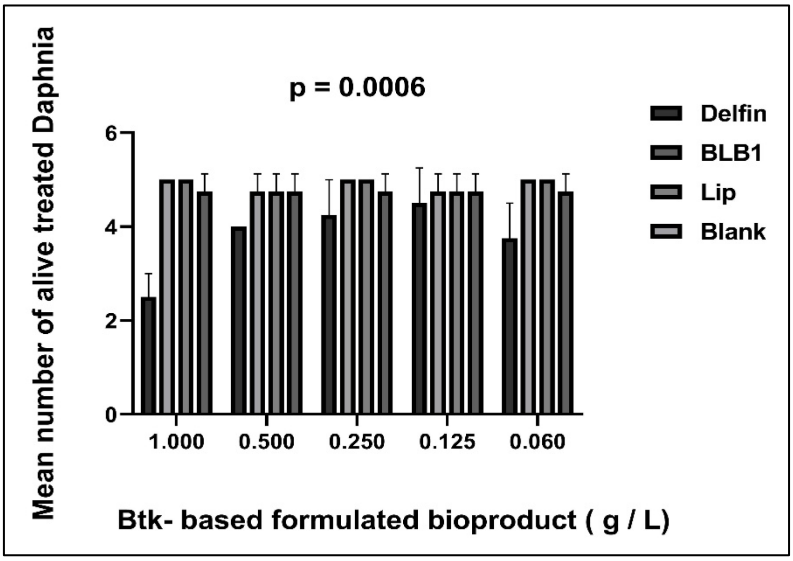

C1	1 g/L
C2	0.5 g/L
C3	0.25 g/L
C4	0.125 g/L
C5	0.06 g/L

Figure S.3. Viability of the treated D. magna after exposition to various Btk-biopesticides formulated whole products, tested at different concentrations (Endpoint assessment at 48 h of alive treated Daphnia). Data expressed relative to mean value. Bars represent the mean ± SE. The significance has been assessed using one-way ANOVA with Dunett test.
